# Dry ginger and Schisandra chinensis modulate intestinal flora and bile acid metabolism to treatment asthma

**DOI:** 10.3389/fmicb.2025.1541335

**Published:** 2025-03-27

**Authors:** Yantong Yu, Shuang Geng, Chao Bu, Gang Cao, Yanquan Han, Dongmei Xie, Yan Hong

**Affiliations:** ^1^Department of Pharmacy, The First Affiliated Hospital of Anhui University of Chinese Medicine, Hefei, China; ^2^School of Pharmacy, Anhui University of Chinese Medicine, Hefei, China; ^3^College of Pharmacy, Zhejiang Chinese Medical University, Hangzhou, China; ^4^School of Integrated Chinese and Western Medicine, Anhui University of Chinese Medicine, Hefei, China

**Keywords:** inflammations, intestinal flora, bile acid metabolism, dry ginger, Schisandra chinensis

## Abstract

**Background and aims:**

*Zingiber officinale* Rosc (Dry ginger) and *Schisandra chinensis* (Turcz.) Baill (Schisandra chinensis) drug pairs (DSP) are often used as drug pairs for the treatment of asthma, and these two traditional Chinese medicines (TCM) are also the core components of multiple TCM. However, its specific pharmacological mechanism needs further research. The aim of this study is to investigate the effects of DSP on intestinal flora and bile acid metabolism in rats with asthma caused by cold, and to provide experimental evidence for its clinical application.

**Materials and methods:**

Sixty male rats are divided into five groups, 12 rats per group. Except for control groups, other groups of rats use the method of “abdominal injection of “OVA + ice water swimming” to establish cold cough rats models. After the administration cycle is over, an optical microscope count method is used to detect eosinophils (EOS) and neutrophils (Neu) in bronchoalveolar lavage fluid (BALF); Enzyme-Linked Immunosorbent Assay (ELISA) method detects Immunoglobulin E (IgE), Interleukin-13 (IL-13), Interleukin-4 (IL-4), Tumor Necrosis Factor-α (TNF-α), Interferon-γ (INF-γ); Western Blot (WB) and Reverse Transcription-Polymerase Chain Reaction (RT-PCR) analysis was used to detect Forkhead box protein P3 (Foxp3) and Transforming Growth Factor-beta 1 (TGF-β1) proteins expression. In addition, Using 16S rDNA sequencing reveal the role of intestinal flora in asthma and the effect on the gut microbiome after DSP treatment. We also examined Farnesoid X Receptor (FXR) proteins expression, and finally used ultra performance UPLC-MS/MS to analyze bile acids (BAs) contentin in rats.

**Results:**

DSP inhibits asthma inflammation. It alleviates inflammatory factors, suppresses the inflammatory response in asthmatic rats by regulating FOXP3 and TGF-β1 in Treg cells, and reduces tissue damage. After DSP treatment, intestinal flora changed: harmful bacteria like *Streptococcus* decreased, while beneficial bacteria such as *Candidatus - Arthromitus* and *Ligilactobacillus* increased. These changes can be potential markers for DSP-intervened asthma. Also, DSP increased FXR protein expression and changed the bile acid spectrum: Deoxycholic acid (DCA) increased, allocholic acid (ACA), glycolithocholic acid (GLCA), glycochenodeoxycholic acid (GCDCA) andglycoursodeoxycholic acid (GUDCA) decreased.

**Conclusion:**

This study has preliminarily revealed that DSP has the effect of alleviating inflammation levels, also regulate the expression of FOXP3 and TGF-β1 proteins, and has an impact on the gut microbiota and bile acid metabolism, thereby exerting an improving effect on asthma and providing a reference for the clinical application of traditional Chinese medicine in the treatment of asthma.

## 1 Introduction

Asthma is a chronic inflammatory disease characterized by airway hyperresponsiveness and reversible airflow obstruction. Studies have demonstrated that the intestinal flora accounts for 78% of the total microbiota of the human body (Lee et al., 2021). Several studies have shown that the intestinal flora is directly related to the development of several human diseases, such as metabolic diseases: hyperlipidemia, diabetes, and asthma (Gao et al., 2020). In addition, the Swedish schola (Abrahamsson et al., 2014) found that significant differences in intestinal flora began to appear in asthmatic newborns in the first week of life compared to healthy newborns.

Bile acids are important components of bile, synthesized from cholesterol and stored in the gallbladder, and can be structurally divided into free bile acids, including cholic acid (CA), chenodeoxycholic acid (CDCA), glycocholic acid (GCA) (Jin, 2019; Wu et al., 2018). It was found that tauroconjugated bile acids were significantly more effective than free bile acids in relieving cough and resolving sputum and asthma (Hu and Shi, 2001; Si, 2002; Zhang et al., 2016), directly dilating the bronchi, counteracting tracheal smooth muscle spasm, and reducing the number of coughs within 3 min. Some studies have shown that Interactions between bile acids and intestinal flora may be directly involved in the diseases mechanism (Guo et al., 2022). In contrast, the modifying effects of intestinal flora on bile acid molecules include three types, debinding, differential isomerization, and dehydroxylation, which can regulate the size of the bile acid pool and the ratio of each bile acid component (Xiong, 2023).

In traditional Chinese medicine (TCM), there is no exact corresponding name for cough variant asthma, and it is mostly categorized as “cough.” The diagnostic and therapeutic guidelines for cough (2015), for the first time, includes the treatment of chinese medicine, It is also believed that cold irritation of the lungs is one of the main causes of asthma.

DSP can benefit the lung, calm asthma and cough, resolve phlegm, and cure cough with wonderful effect. Zingiberis Rhizoma (Dried ginger) is the rhizome of *Zingiber officinale* Roscoe, with pungent flavor and hot nature, which has the efficacy of warming the middle and dispersing the cold, restoring the yang to pass through the veins, and warming the lungs (Zhuo et al., 2024); Schisandrae Chinensis Fructus (Schisandra chinensis) is obtained from the dried mature fruit of *Schisandra chinensis* (Turcz.) Baill, It works by warming the lungs and alleviating asthma and cough (Ma et al., 2009).

This study employed a comprehensive approach using 16S rDNA gene sequencing to analyze changes in the gut microbiota of an asthmatic rat model and the effects of drug intervention. The study found that DSP can activate FXR, leading to the measurement of 19 bile acids and monitoring of Treg cell differentiation. To better understand the functional correlation between gut microbiota changes and metabolic alterations, a correlation analysis was conducted between the altered microbiota and disturbed metabolites. The study aim of the study was to investigate the impact of DSP on gut microbiota, thereby affecting bile acid metabolism, regulating pulmonary Treg differentiation, and inhibiting the progression of asthma.

## 2 Materials and methods

### 2.1 Sample preparation

In this study, the water extracts of dried ginger and Schisandra chinensis (9:5) were used. Dried ginger and Schisandrae Chinensis were obtained from Anhui Huqiao Pharmaceutical Co., Ltd. (batch number: 2200203057). After weighing, they were decocted with 10 times water, then decocted again with 8 times water, the filtrates were combined, and concentrated using a rotary evaporator.

### 2.2 Chromatographic fingerprint analysis

Each batch of 10 batches of dried ginger and Schisandra chinensis (Supplementary Table S1), weighing, heating and ultrasonic (150 W, 40 Hz) for 40 min, cooled, weighed, added methanol to make up the weight, shaking, filtration, centrifugation at 12,000 rpm for 12 min before the determination. Analysis was carried out on a Waters ACQUITY UPLC BEH C18 column (2.1 mm × 100 mm, 1.7 μm, Waters, United States); Column temperature 30°C; The mobile phase acetonitrile (A)—0.1% phosphoric acid aqueous solution (B) and the flow rate was 0.2 mL/min. The gradient elution program was as follows: 3–20% A from 0 to 3 min; 20–35% A from 3 to 4 min; 35–40% A from 4 to 5 min; 40–46% A from 5 to 6 min; 46% -5 5% A from 6 to 7 min; 55–55% A from 7 to 14 min; 55–90% A from 14 to 18 min; 90–90% A from 18 to 23 min; 90–100% A from 23 to 26 min; 100–3% A from 26 to 28 min; 3–3% A from 28 to 30 min; Detection wavelength: 280 nm; Injection volume: 2 μL.

### 2.3 Animals experiments

The rats were obtained by “OVA+ice water swimming” to establish cold cough rats models, following the previously described methodology (Ran et al., 2019). Sixty Sprague–Dawley (SD) male rats (200 ± 20 g), were purchased from Henan Province Experimental Animal Quality, Supervision and Monitoring Station and were raised at in the laboratory Anhui University of traditional Chinese medicine.

Five rats groups (*n* = 12) were created: Control, Model, DSP (L/H), and Dexamethasone (DXM) groups. The clinical dosage of DSP is typically 14 g/d, for an adult weighing 60 kg (Lang, 2013). The equivalent dosage for rats, calculated based on the body surface area (weight) ratio between humans and rats, is 1.47 g/kg. The DSP (L/H) groups were established according to the principle that the equivalent dosages are 1 and 4 times the adult dosages (1.47, 5.88 g/kg/d), respectively (Li, 2006). The DXM group were given at a final dosage of 0.75 mg/kg.

In addition to the control group, the remaining 48 SD rats were injected intraperitoneally with 1.0 mL of OVA mixture (10 mg/mL ovalbumin and 100 mg/mL aluminum hydroxide) on days 1 and 8, respectively, to induce the preparation of asthma rat model; Starting from day 15, the rats were stimulated by nebulization with a 2% OVA solution every day for 30 min for 15 days. After the rats were anesthetized by intraperitoneal injection of 0.3% pentobarbital sodium solution (30 mg/kg). 3 mL of saline was inhaled from a 5 mL syringe, the neck and chest of the rats were dissected, the trachea and one lobe of the lung were ligated, and then a syringe needle was inserted into the upper end of the trachea, and saline was slowly injected into the trachea of the rats for back-drawing and lavage, which was repeated three times. The collected alveolar lavage fluid was centrifuged at 1,500 rpm for 10 min, and the supernatant and precipitate were separated separately. After obtaining the rat BALF, the middle lobe of the right upper lung of the rat was retained and fixed in 10% neutral formalin for 24 h, pending analysis. Animal Ethics Number AHUCM-rats-2022093.

### 2.4 Histopathological analysis

After obtaining the rats balf, leave the rats lung and soak 24 h in 10%, and Observe the tissue structure under the microscope.

### 2.5 Inflammatory cell count

The Balf was centrifuged at 3,000 rpm for 10 min, and the supernatant was stored in a refrigerator at −80°C after centrifugation. The resulting cell precipitates were fixed on slides for richter staining, and the proportions of eosinophils and neutrophils were counted under light microscope, respectively.

### 2.6 Determination of inflammatory factors

The concentrations of IgE, IL-13, IL-4, TNF-α, and INF-γ in the serum and Balf of different groups of mice were measured by ELISA method according to the instructions on the kit, respectively.

### 2.7 Gut microbiota analysis

Firstly, rat feces were extracted and the extracted DNA was examined. The concentration of DNA was measured by fluorescence Spectrophotometer (Invitrogen, California, United States), measuring the absorbance value of DNA at 260 nm and 280 nm. The quality of DNA was detected by 1% agarose gel electrophoresis. The concentration of DNA solution was adjusted. After the amplification of the 16 s rRNA gene variable region, gel recovery and purification were performed: the target bands were cut and recovered to obtain the purified samples; then each sample was quantified using a (BioTek, Vermont, United States) enzyme marker. Finally, the standard Illumina TruSeq DNA library preparation procedure was used to construct the required online libraries.

### 2.8 Western blotting

Precisely weigh out 50 mg of lung and intestine tissue, add RIPA (Servicebio, Wuhan, China) to lyse the tissue, which were subsequently quantified using the BCA (Servicebio, Wuhan, China) method. Then, subject the protein sample to SDS-PAGE electrophoresis. The membrane was incubated at room temperature for 3 h in the primary antibody incubation solution and subsequently incubated overnight at 4°C. After washing, detect the protein signal using a chemiluminescent substrate and quantify the signal using image analysis software. The primary antibodies used were as follows: anti-FXR (rabbit; dilution: 1:1000; Catalog number: 77G1724, Affinity Biosciences, China), anti-FOXP3 (rabbit; dilution: 1:1000; Catalog number: AF6544, Affinity Biosciences, China), anti-TGF-β1 (rabbit; dilution: 1:1000; Catalog number:AF1027, Affinity Biosciences, China), and anti-β-actin (rabbit; dilution: 1:3000; Catalog number: AF7018, Affinity Biosciences, China). The following secondary antibodies were used: goat anti-rabbit IgG (Catalog number: S0001, 1:3000 dilution; Affinity Biosciences, China).

### 2.9 RT-PCR

Weigh out 100 mg each of lung and intestine tissue, homogenize them thoroughly using a tissue grinder, and then add 1 mL of the RNA extraction reagent Trizol for lysis. Subsequently, convert the RNA into cDNA using reverse transcriptase. Through cycles of denaturation, annealing, and extension, accurately replicate the target DNA sequences. After amplification, evaluate gene expression. The primer sequences are shown in (Supplementary Table S2).

### 2.10 Bile acid analysis

The standard and internal standard solutions with concentrations were prepared separately. Then, weigh 25 mg of rat ileum contents, add 1,000 μL of extraction solution (methanol: acetonitrile:water = 2:2:1), containing 100 μL each of 5,000 ng/mL CA-D4, LCA-D4, and GCDCA-D4 internal standards; vortex and mix for 30 s; add 2 small steel balls, grind at 65 Hz for 3 min, and sonicate in an ice-water bath (100 Hz, 5 min). The samples were centrifuged at 4°C for 15 min at 13,000 rpm; the samples were aspirated into an LC injection vial.

### 2.11 Statistical analysis

The experimental data were analyzed using SPSS 22.0 statistical software, and one-way ANOVA was used for comparison of multiple sample means. If the experimental data were non-normally distributed, the rank sum test (Kruskal-wallis h test) was used.

## 3 Results

### 3.1 Fingerprint analysis

**Figure 1:**
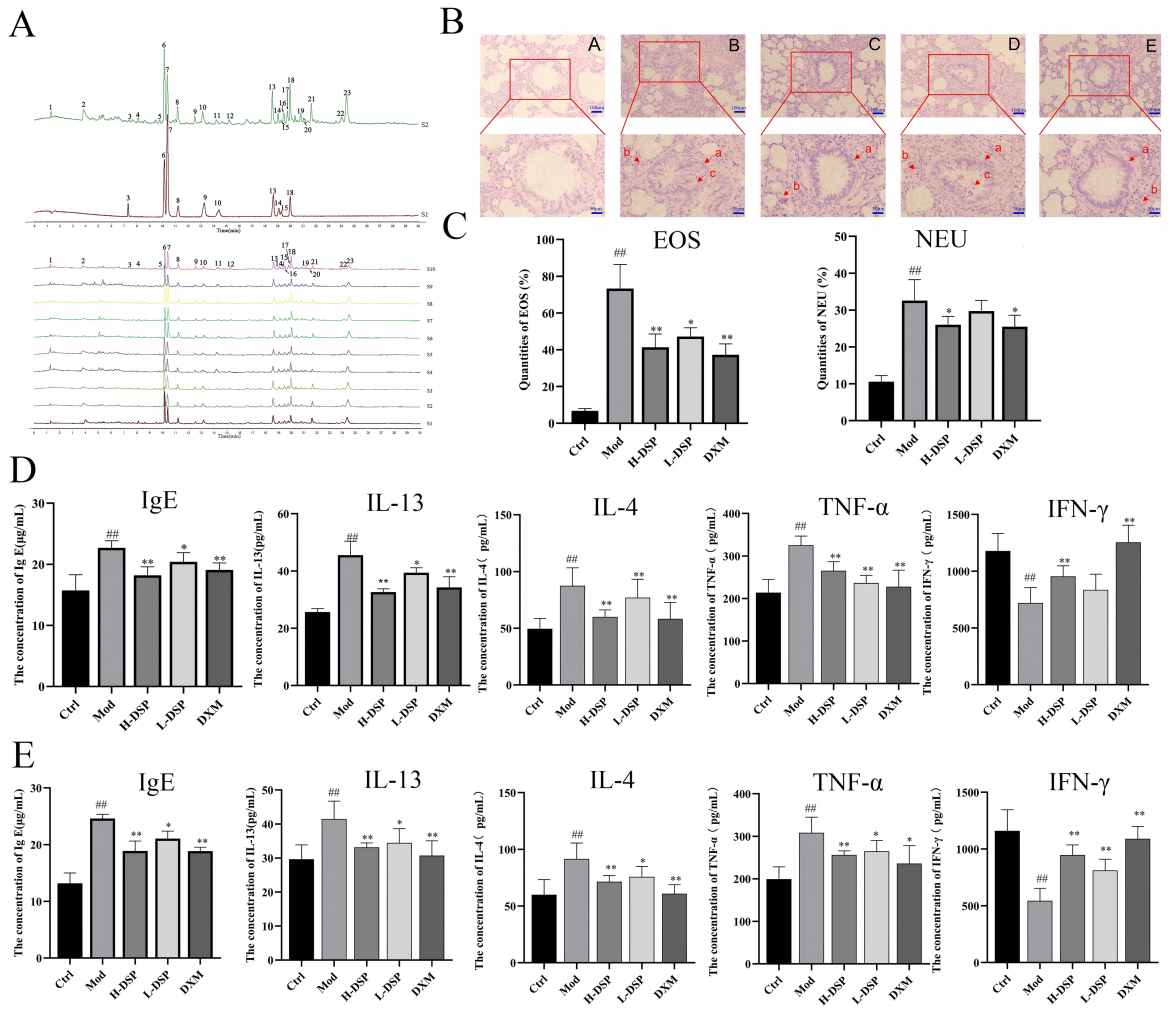
DSP inhibits inflammation in asthmatic rats. **(A)** DSP fingerprinting. **(B)** HE staining of lung tissues of rats in each group. **(C)** Classification of inflammatory factors in each group of rats. **(D)** Detection of inflammatory cell in BALF in rats in each group. **(E)** Detection of inflammatory cell in serum in rats in each group (Compared with the control: ^#^*p* < 0.05, ^##^*p* < 0.01; Compared with the model group: *p < 0.05, **p < 0.01, *n* = 12).

The UPLC profiles of the obtained DSP were sequentially imported into the chinese medicine chromatographic fingerprint similarity evaluation software 2012 (version A) (Figure 1A). The results showed that there were 23 peaks, of which 10 peaks were identified. The peaks 3, 6, 7, 8, 9, 10, 13, 14, 15, and 18 were zingerone, 6-gingerol, Schisandrol A, Gomisin A, 8-gingerol, 6-Shogaol, 10-Gingerol, Schizandrin A, 8-Shogao, Schizandrin B.

### 3.2 DSP alleviates the inflammatory response

It can be seen from (Supplementary Table S3) that in the first week of modeling, there was no difference in body weight among rats in each group; by the fourth week, the body weight of rats in the model group was significantly lower than that of rats in the control group (*p* < 0.01), and the DSP group showed a significant trend of recovery. The pathological changes in lung tissue were assessed using HE staining. In the model group showed increased bronchial wall thickness (Figure 1B, (B): a), narrowed lumen, irregular shape, increased mucus secretion in the bronchial lumen (Figure 1B, (B): c), and a large amount of inflammatory cell infiltration around the lumen (Figure 1B, (B): b). After DSP treatment, there were also a small amount of inflammatory cell infiltration around the bronchial tubes (Figure 1B, (C, D, E): b), but significantly less than that in the model group, with the thickness of bronchial tube wall significantly thinned (Figure 1B, (C, D, E): a), and the secretion of mucus in the lumen of the bronchial tubes significantly reduced. Compared with the lung tissue sections of H-DSP group, the thickness of the bronchial tube wall increased in the L-DSP group (Figure 1B, (D): a), and the mucus secreted in the tube lumen increased (Figure 1B, (D): c).

### 3.3 DSP reduces the number of inflammatory cells in the BALF of asthmatic rats

In this study, we assessed the impact of drug intervention on asthmatic inflammatory responses by analyzing the changes in eosinophil and neutrophil counts before and after treatment (Figure 1C). The results showed that in the model group, the numbers of both types of granulocytes significantly increased. After treatment, the counts of both cell types decreased. Additionally, it is noteworthy that the therapeutic effect of DSP is positively correlated with the dose.

### 3.4 DSP reduces inflammation in asthma serum and BALF

In serum and BALF (Figures 1D,E), compared with the control group, the levels of IgE, IL-13, IL-4, and TNF-α in the model group were significantly increased (*p* < 0.01), while the level of IFN-γ was decreased (*p* < 0.01). After drug treatment, these cytokine levels approached those of the control group.

### 3.5 DSP recovered the disordered intestinal flora in asthmatic rats

**Figure 2:**
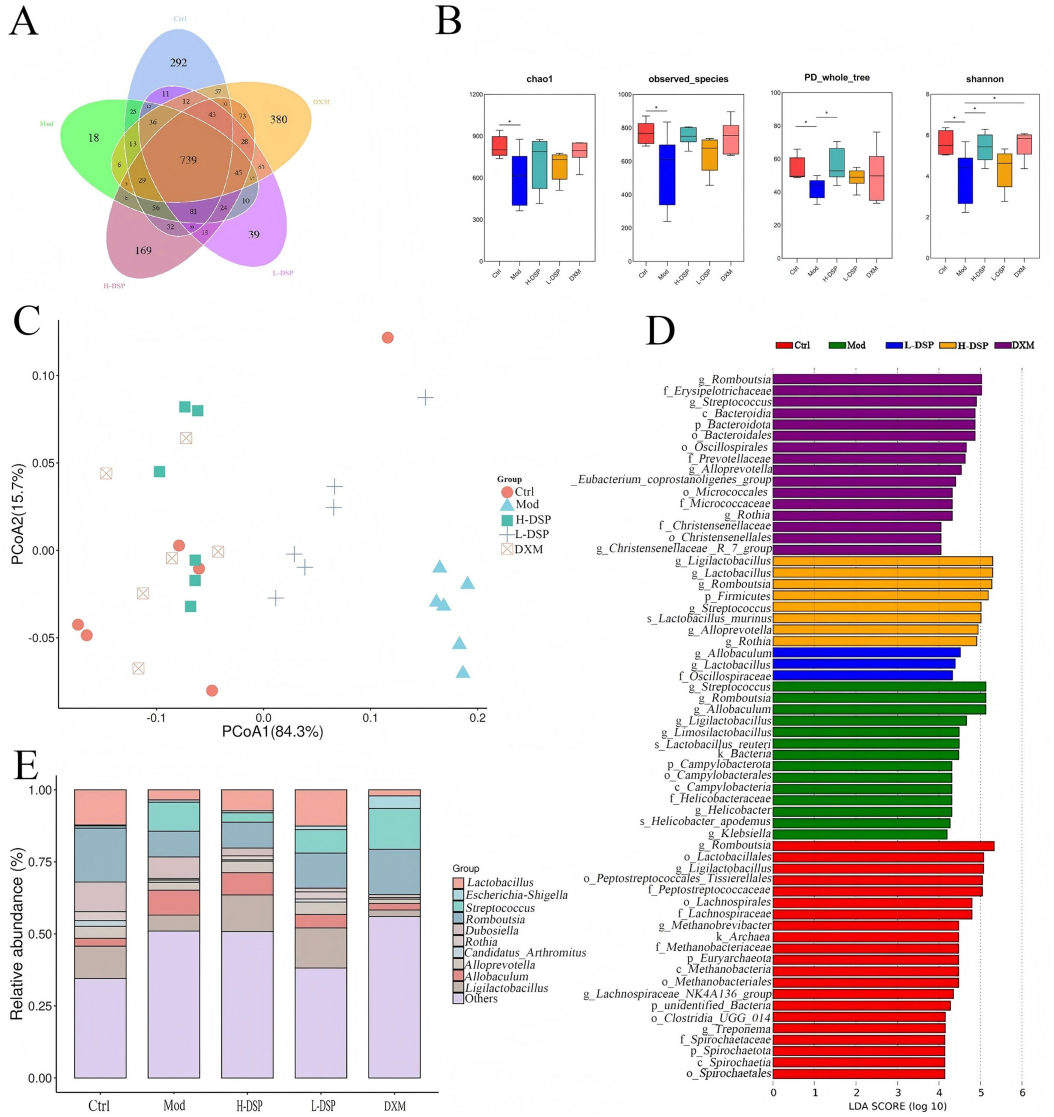
Effect of DSP on intestinal microorganisms in the feces of rats with asthma. **(A)** The number of OTUs in the intestinal flora of rats in each group. **(B)** The richness of the intestinal flora was assessed by the chao1 index and the observed species index, and the diversity of the intestinal flora was assessed by the PD whole-tree index and the Shannon index. **(C)** Beta-diversity of bacterial communities represented by PCOA plots. **(D)** Potential identification of gut microbial taxa in control, model, pharmaceutical and dexamethasone groups based on LEFSE analysis. **(E)** Relative abundance of genus levels of intestinal flora in each group of rats (*p < 0.05, *n* = 6).

OTU is a basic unit in microbial community analysis. After clustering a total of 2,404 OTUs were obtained, with 739 being common to all five groups (Figure 2A). The changes in gut microbiota diversity were determined by α-diversity estimators. The PD whole tree index and Shannon index indicated significant differences in community richness and diversity between the model and control groups (*p* < 0.05) (Figure 2B). The Chao index revealed a marked decrease in intestinal microbiota richness after modeling. DSP treatment slightly restored the richness of the intestinal microbiota.

β-diversity of intestinal microbiota across groups was analyzed using Principal Coordinate Analysis (PCoA). It shows that PCoA indicated significant changes in the intestinal microbiota composition of model group rats (Figure 2C).

LEfSe analysis, with a default LDA score cut-off of 4, and the results of the intergroup relative analysis showed distinct microbial communities between the control, model, DSP, and DXM groups (Figure 2D,E). The control group was enriched with *Romboutsia, Ligilactobacillus*, and *Lactobacillus*. *Streptococcus* was more abundant in the model group.

### 3.6 Relative abundance analysis of dominant species between groups

**Figure 3:**
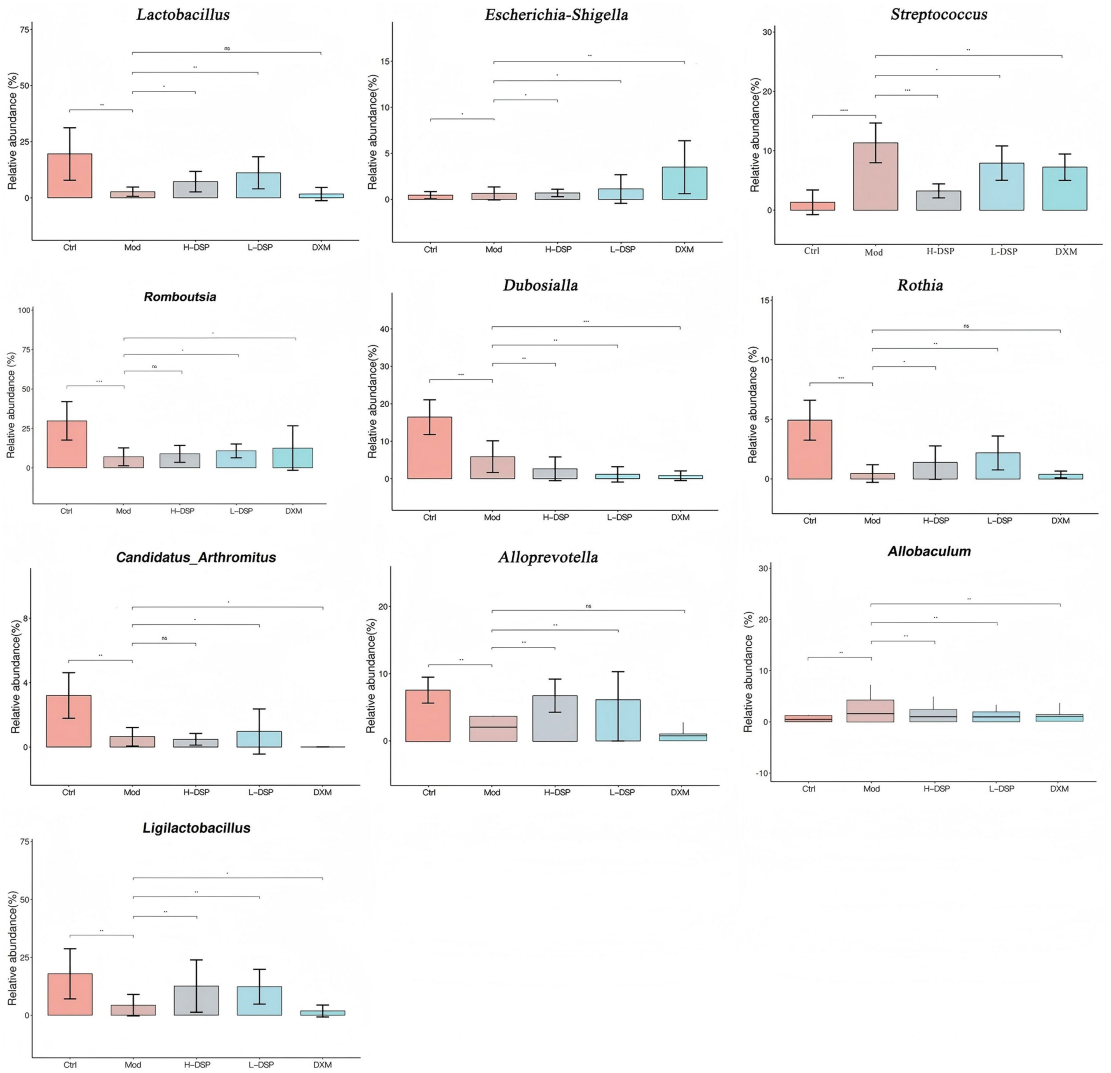
Relative abundance of genus levels of intestinal flora in each group of rats (Compared with the control: ^#^*p* < 0.05, ^##^*p* < 0.01; Compared with the model group: *p < 0.05, **p < 0.01, *n* = 6).

This study further investigated the trends of 10 bacterial genera that showed significant changes before and after administration (Figure 3). Compared with the model group, the control group showed higher levels of *Dubosiella*, *Candidatus_Arthromitus*, *Romboutsia*, and *Rothia*. *Streptococcus* significantly increased after modeling (*p* < 0.05) and significantly decreased after treatment (*p* < 0.05). This result is consistent with the previous study.

### 3.7 DSP activates bile acid receptor FXR

**Figure 4:**
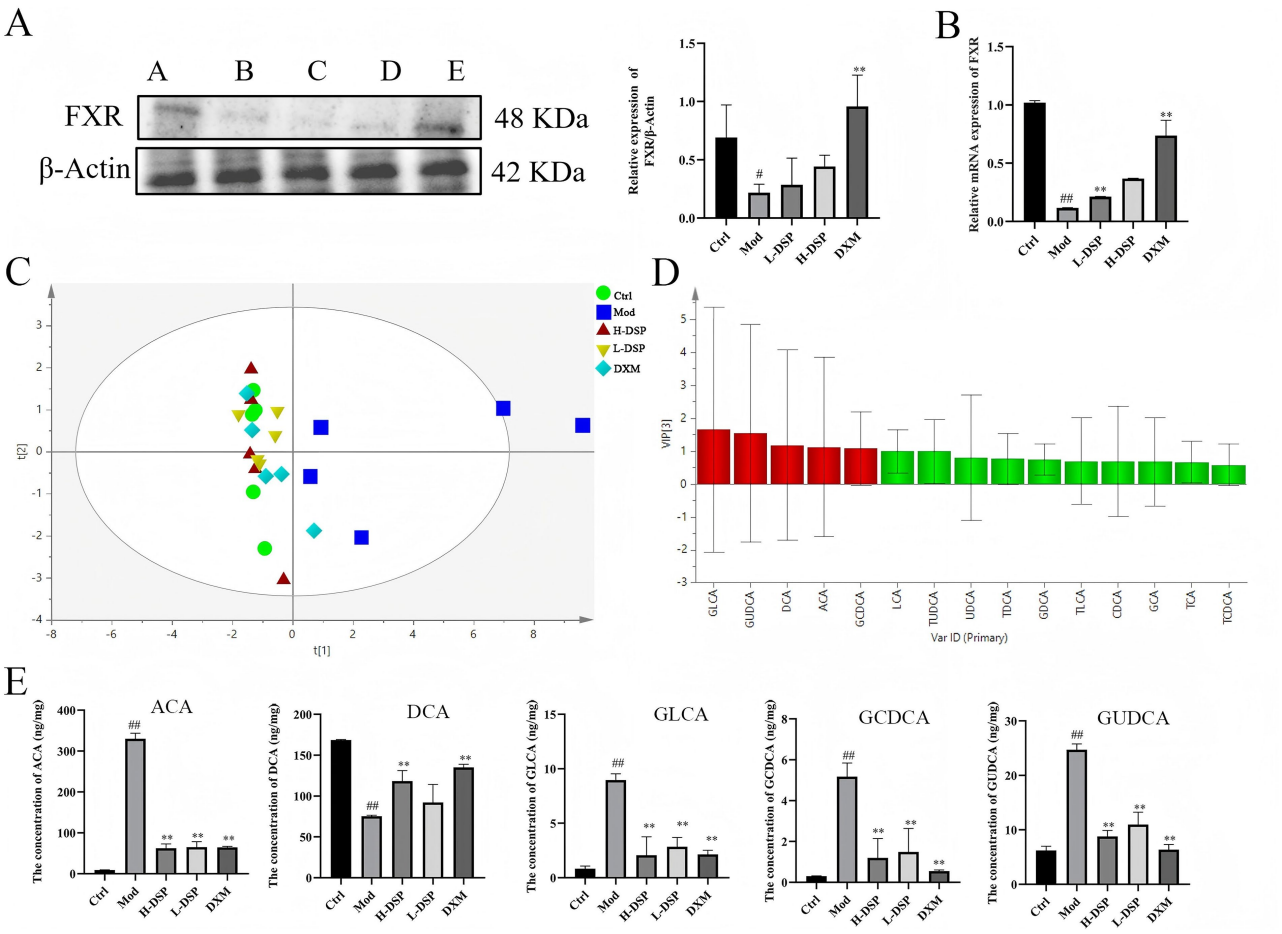
Effect of DSP on fecal bile acids in asthmatic rats. **(A)** FXR expression was reduced in the model group (control images are re-used for illustrative purposes, *n* = 3). **(B)** mRNA expression of FXR (*n* = 6). **(C)** OPLS-DA scores of bile acids in each group. **(D)** Analysis of fecal bile acids in rats: differential marker VIP plot. **(E)** Difference in bile acid content in rat feces (compared with the control: ^#^*p* < 0.05, ^##^*p* < 0.01; compared with the model group: *p < 0.05, **p < 0.01, *n* = 6).

Farnesoid X receptor (FXR), a crucial nuclear receptor, plays an essential role in regulating bile acid metabolism, preserving intestinal mucosal barrier integrity, and fostering liver cell regeneration. The intestinal flora influences the composition of bile acids, thereby affecting FXR signaling pathways. Western blot and RT-PCR results of colonic tissue revealed that FXR protein expression was significantly reduced in the model group (*p* < 0.05; *p* < 0.01) (Figures 4A,B). Treatment of asthmatic rats with DSP increased FXR protein levels.

### 3.8 DSP modulated the fecal bile acids in asthmatic rats

Previous research found that DSP can activate FXR protein expression of the bile acid receptor. The FXR receptor prompts further investigation into the impact of FXR activation on bile acid metabolism. Therefore, we detect 19 bile acid standards by LC–MS/MS, and the results showed great separation of the bile acids (Supplementary Figure S1), furthermore, we conducted a evaluation of the standard curve, determination of the linear range, assessment of precision, monitoring of stability, and testing of spike recovery rate (Supplementary Tables S4–S7). The results all indicated the reliability of the instruments and analytical methods used. Then, we analysis on the bile acid data. It shows that the model group is the farthest from the control group, while the DSP group is closest to the control group, indicating that DSP affects bile acid metabolism. Finally, we used OPLS-DA to analyze the chromatographic peaks and obtained the VIP plot (Figure 4C), selecting bile acids with VIP > 1 as differential bile acids, which are GLCA, GUDCA, DCA, ACA, and GCDCA (Figure 4D). The subsequent analysis content (Figure 4E), revealed that compared to the control group, the contents of ACA, GLCA, GCDCA, and GUDCA in the model group significantly increased (*p* < 0.01), while the content of DCA significantly decreased (*p* < 0.01). The DSP group was able to regulate bile acids to approach the levels found in the control group plot.

### 3.9 Correlation analysis of intestinal flora and bile acid metabolism

**Figure 5:**
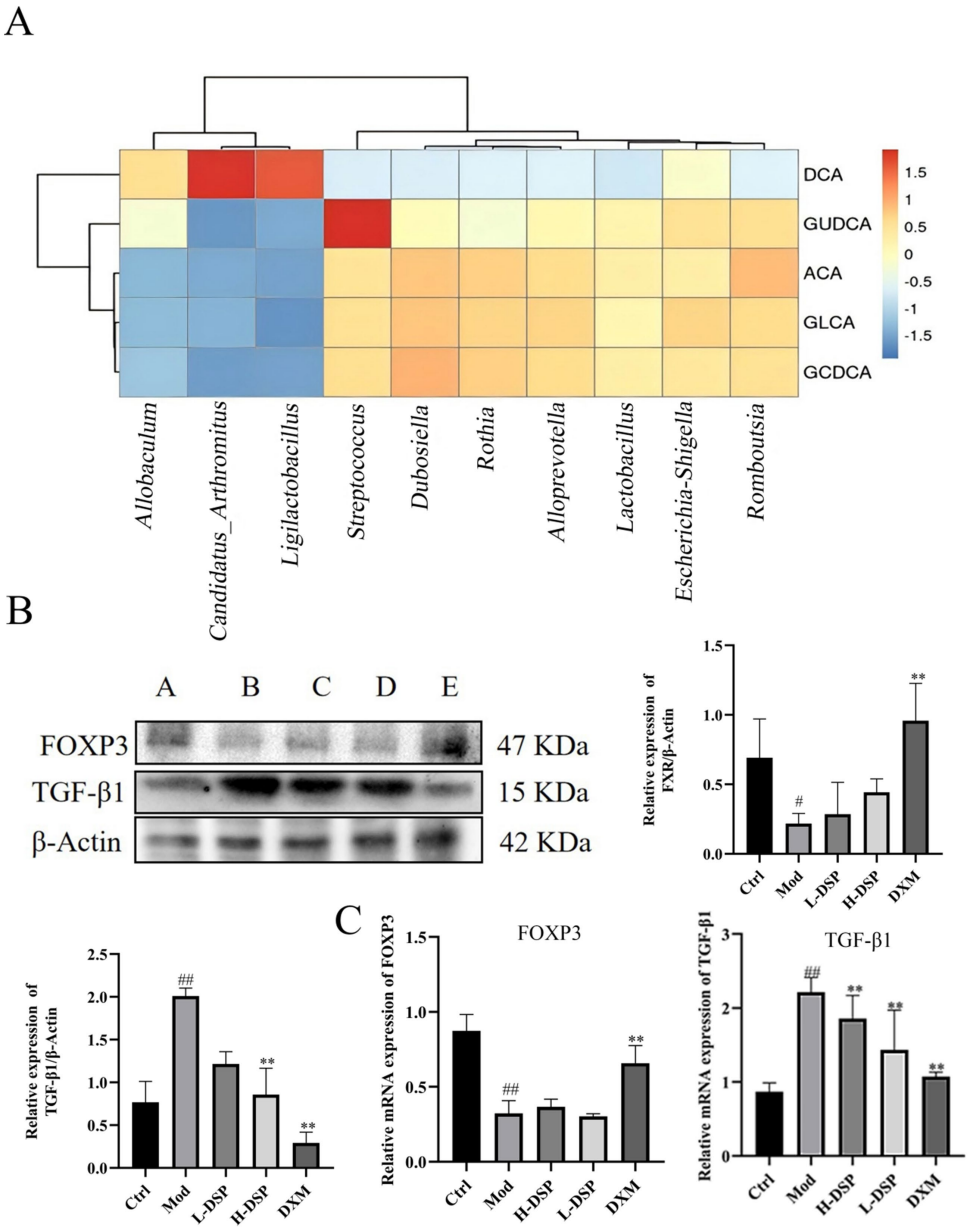
**(A)** Correlation analysis of intestinal flora and bile acid metabolism. **(B)** FOXP3 and TGF-β1 protein expressions (control images are re-used for illustrative purposes, *n* = 3). **(C)** mRNA expression of FOXP3 and TGF-β1 (compared with the control: ^#^*p* < 0.05, ^##^*p* < 0.01; compared with the model group: *p < 0.05, **p < 0.01, *n* = 6).

To explore the functional link between intestinal flora changes and metabolic alterations, we performed Spearson correlation analysis on the altered flora and affected metabolites, revealing a strong correlation. The results were shown in the heatmap where the color range of red represents a positive correlation, and the color range of blue represents a negative correlation (Figure 5A). *Candidatus_Arthromitus*, and *Ligilactobacillus* are positively correlated with DCA, and these metabolites are enriched in the Con and DSP groups. At the same time, *Streptococcus*, which is enriched in the model group, are positively correlated with GUDCA.

### 3.10 DSP regulates Treg cells in the lung tissue of asthmatic rats

Metabolites of bile acids can regulate the function of various immune cells, including Th17 and Treg cells, thereby promoting immune system balance and reducing the occurrence of inflammatory diseases such as asthma. Treg cells primarily rely on the expression of the transcription factor Foxp3 for their differentiation (Zhang et al., 2023). These cells can produce a range of immunosuppressive factors, such as TGF-β1, to inhibit the activation and function of other immune cells. We detected Foxp3 and TGF-β1 proteins expression in lung tissue. WB revealed that Foxp3 protein levels were significantly decreased (Figure 5B), while TGF-β1 protein expression were significantly increased compared to the control group (Figure 5B). RT-PCR results were consistent with the trends seen in the Western blotting (Figure 5C). These findings indicate that DSP can regulate Treg differentiation in the lungs, reduce the expression of pro-inflammatory factors, and increase anti-inflammatory factors, thereby modulating the immune response and protecting the lung barrier.

## 4 Discussion

Asthma is a special type of asthma because the clinical symptoms are mainly cough, which is not life-threatening but can cause inconvenience to patients. There is currently no specific drug for asthma, so further research is needed for asthma, whose efficacy has been confirmed in clinical practice and is proven and relatively safe in the treatment of asthma (Nguyen et al., 2023). We found that DSP reduced the infiltration of inflammatory cells around the bronchi in asthmatic rats, significantly thinning the bronchial wall and markedly decreasing mucus secretion in the lumen.

After treatment with DSP, the disorder of the intestinal flora in asthmatic rats was improved, and 10 different types of bacteria closely associated with asthma were screened out. *Romboutsia* and *Lactobacillus* are healthful flora (Fijan, 2014; Moreno et al., 2023). The enrichment of these bacterial groups serves as a pathway for anti-obesity activity and can indirectly improve the symptoms of asthma; *Candidatus- Arthromitus*, *Ligilactobacillus* and *Dubosiella* enhances gut immunity and promotes resistance to inflammatory diseases, thereby reducing inflammation caused by asthma. In this study, the relative abundance of these bacteria was low in the model group, and increased to varying degrees after DSP administration. *Streptococcus* are harmful bacteria. In the study, we found that it is differentially reduced in asthmatic rats after administration of the DSP.

FXR is a member of the nuclear receptor family and is primarily expressed in organs such as the liver and small intestine. Bile acids are the natural ligands of FXR, which is why it is sometimes referred to as the bile acid receptor. In our study, we found that DSP affected the expression level of FXR. As a result, we tested the content of bile acids and found that DSP changed bile acid metabolism. It increased the content of DCA and decreased the content of ACA, GLCA, GCDCA, and GUDCA. Bile acids are also involved in the regulation of immune homeostasis and intestinal barrier function, which help maintain immune tolerance and prevent autoimmune reactions (Koutsounas et al., 2012). We found that DSP increased Foxp3 and decreased TGF-β1 protein expressions in rat lung tissue. From this, it can be seen that DSP can maintain bile acid homeostasis, reduce the level of immunosuppressive factor.

These research findings provide reference information for the use of DSP in the treatment of asthma. However, we have only studied the effects of DSP on the intestinal flora and bile acid metabolism, and have not yet explored its metabolic pathways. In subsequent research, we will delve into the key pathways through which DSP regulates bile acid metabolism and verify them through experiments with clinical samples, with a focus on using comprehensive metabolomics analysis to reveal the detailed mechanisms of intestinal flora changes and bile acid transformation.

## 5 Conclusion

This study suggests that DSP can improve the symptoms of model rats and regulate the abundance of gut microbiota and maintain bile acid homeostasis. DSP may alleviate inflammation by increasing the relative abundance of *Candidatus-Arthromitus* and *Ligilactobacillus*, and upregulating the content of DCA; reducing the relative abundance of *Streptococcus* and down-regulating the content of GUDCA. These findings provide experimental evidence for the development of DSP as a potential traditional Chinese medicine for the treatment of asthma.

## Data Availability

The datasets presented in this study can be found in online repositories. The names of the repository/repositories and accession number(s) can be found in the article/Supplementary material.
