# Supplementary figures and images for: Dry ginger and Schisandra chinensis modulate intestinal flora and bile acid metabolism to treatment asthma
Source: Front Microbiol. 2025 Mar 27;16:1541335. doi: 10.3389/fmicb.2025.1541335 (PMC11984949; doi:10.3389/fmicb.2025.1541335)

Figure. S1 Chromatogram of each bile acid


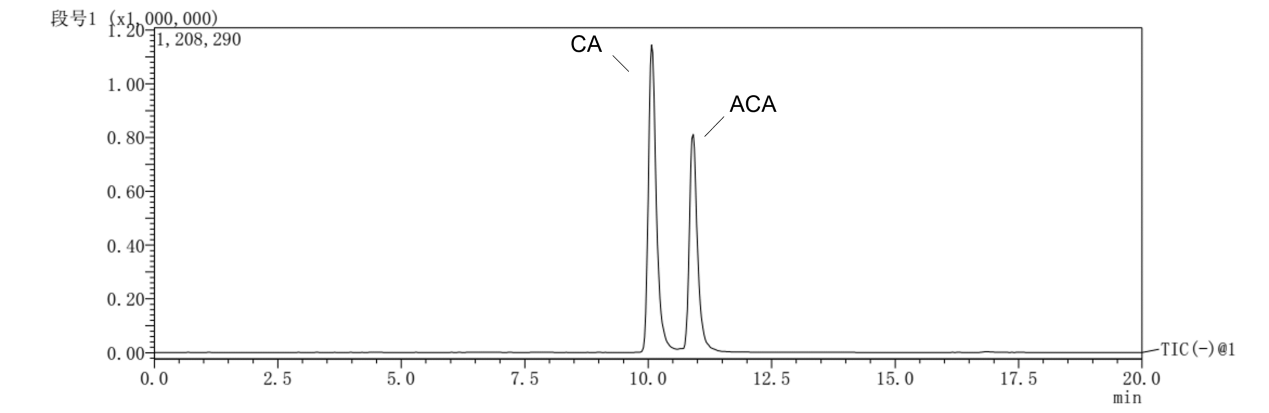


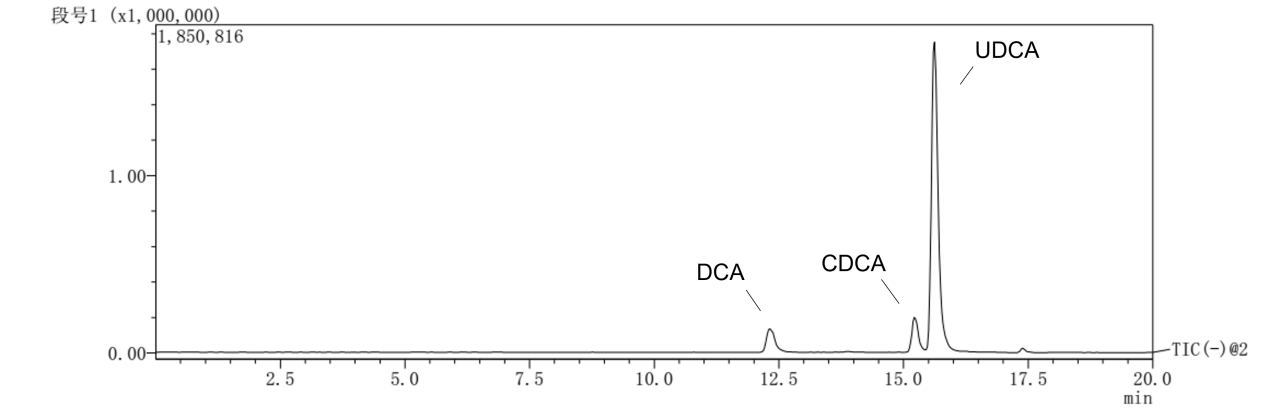


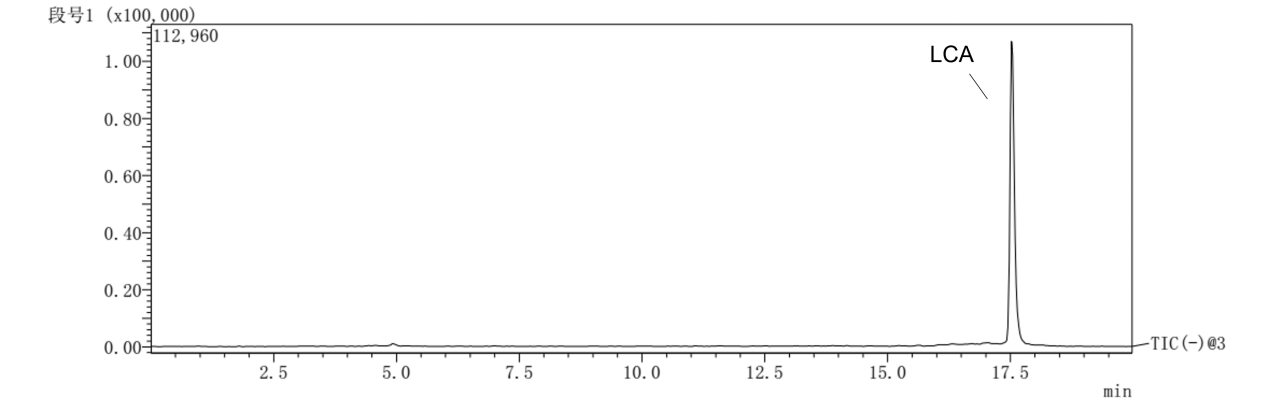


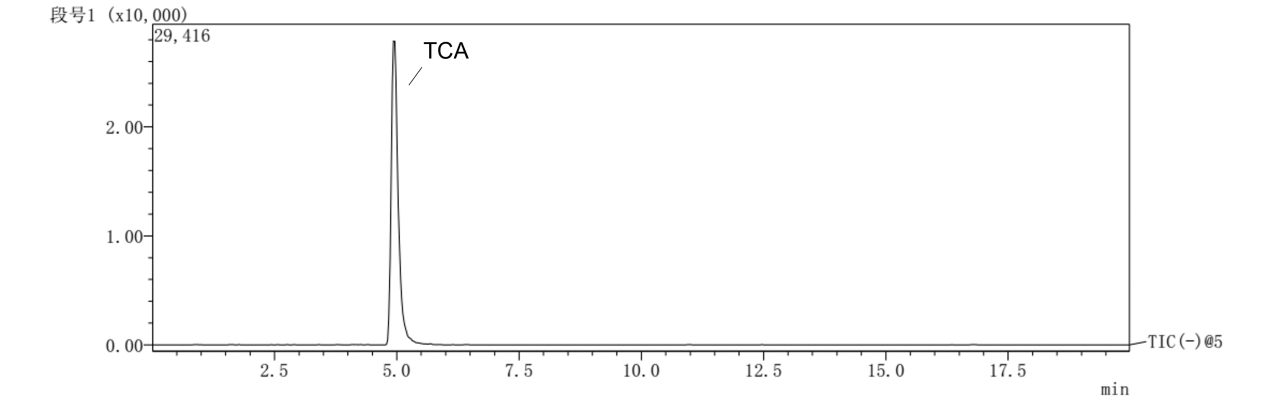


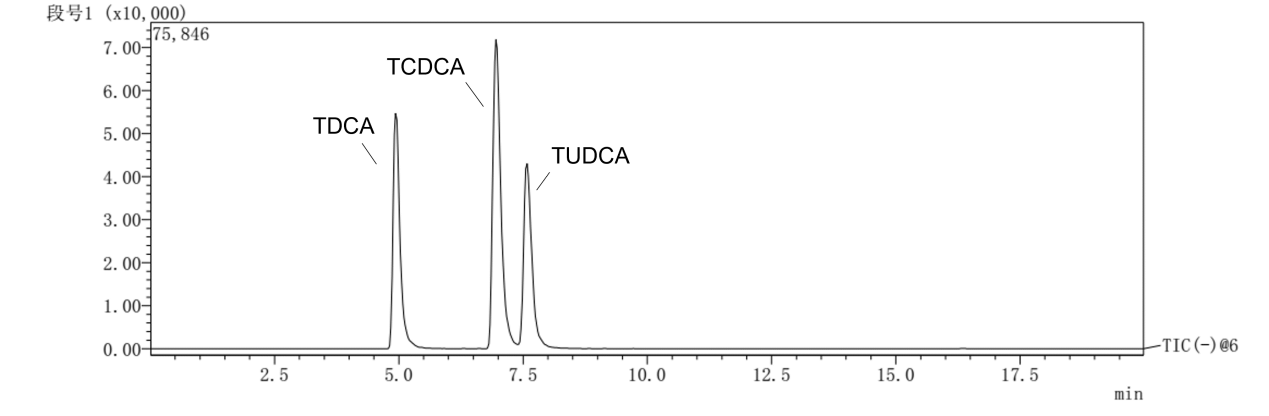


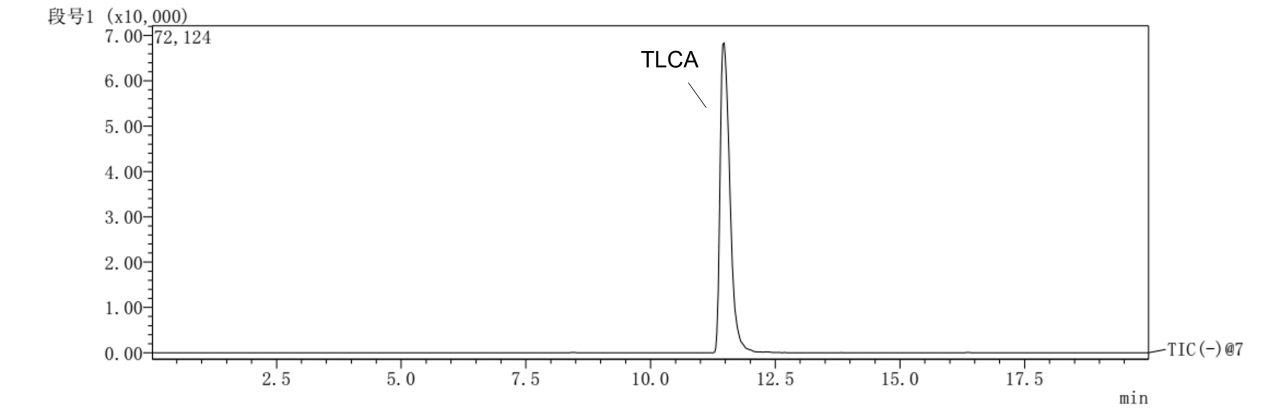


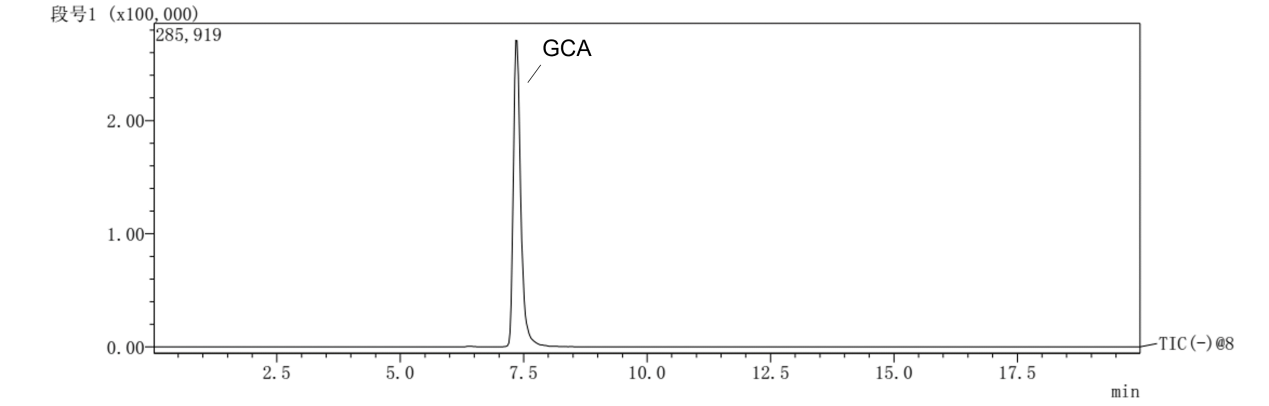


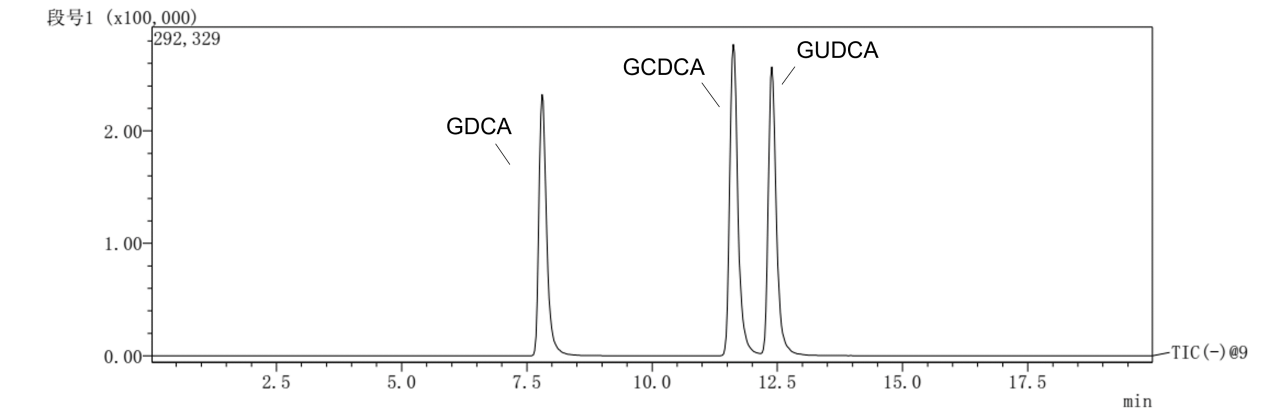


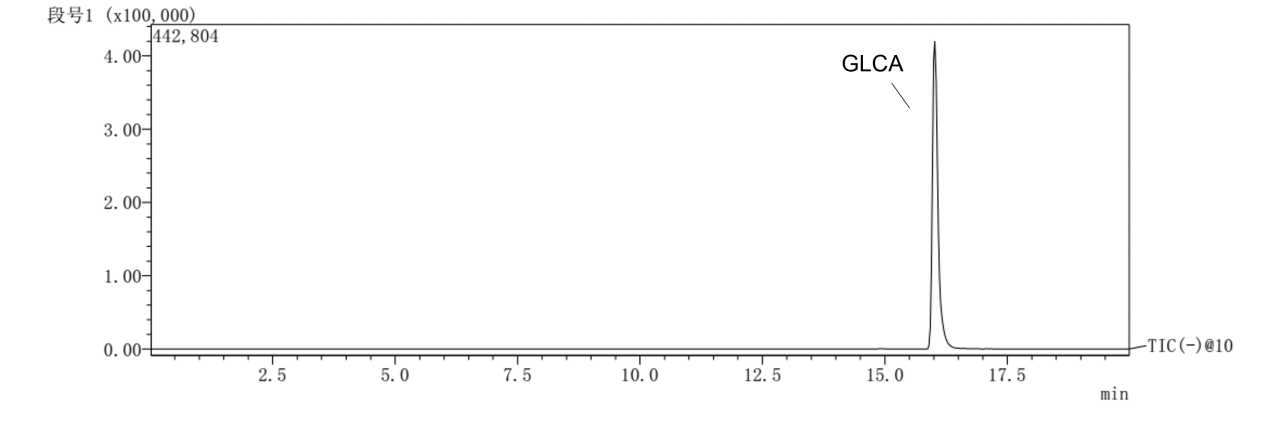


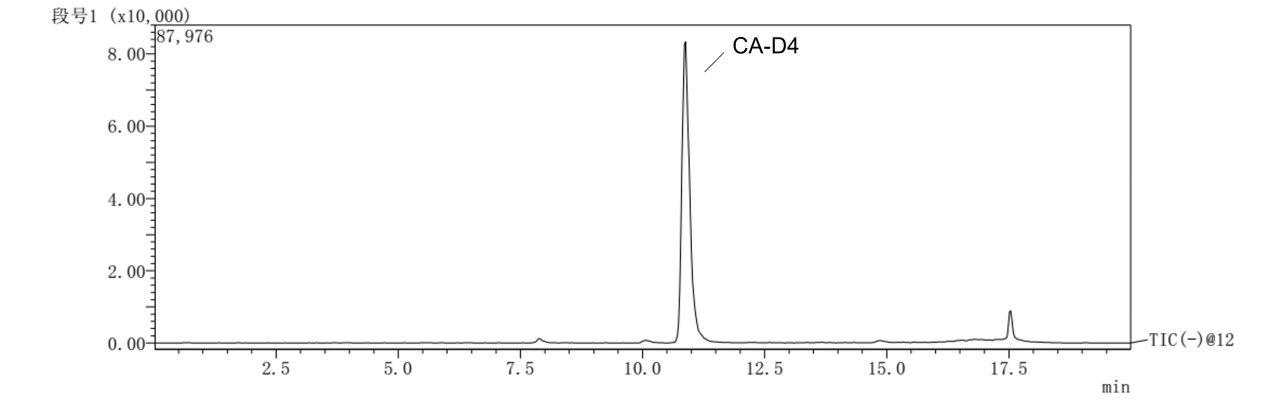


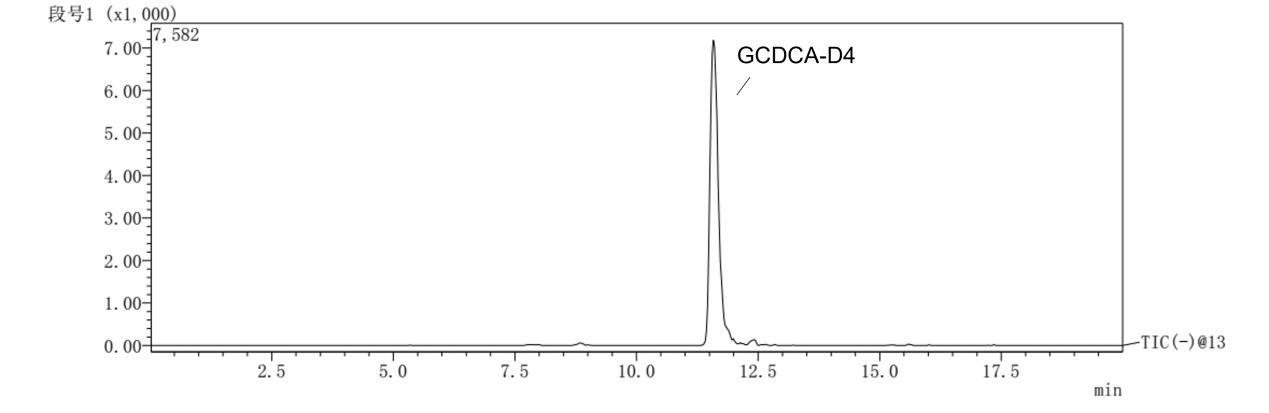


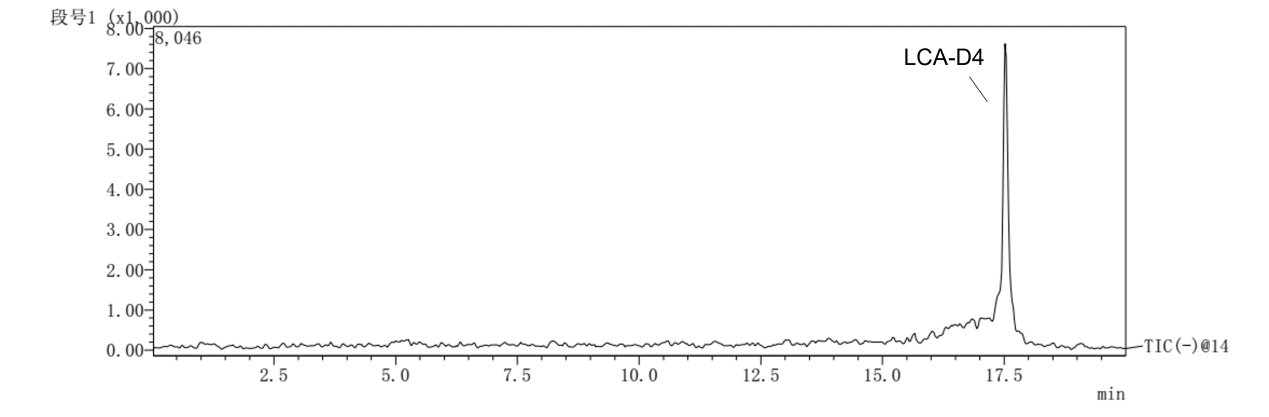

Supplement: Supplementary file 1 [file Supplementary_file_1.docx]
