# Supplementary material for: Dry ginger and Schisandra chinensis modulate intestinal flora and bile acid metabolism to treatment asthma
Source: Front Microbiol. 2025 Mar 27;16:1541335. doi: 10.3389/fmicb.2025.1541335 (PMC11984949; doi:10.3389/fmicb.2025.1541335)
Supplement: Supplementary file 2 [file Table_1.docx]

Table. S1 Origin and batch number of 10 batches of DSP

| No | Dried ginger Origin | Dried ginger Batch Number | Schisandra chinensis  Origin | Schisandra chinensis  Batch Number |
| --- | --- | --- | --- | --- |
| S1 | Sichuan | 20191012 | Anhui | 20191110 |
| S2 | Sichuan | 20190926 | Yunnan | 20190306 |
| S3 | Sichuan | 20181220 | Yunnan | 20170901 |
| S4 | Shandong | 20190921 | Shandong | 19070201 |
| S5 | Shandong | 20181220 | Zhejiang | 20181220 |
| S6 | Liaoning | 20200531 | Heilongjiang | 20210607 |
| S7 | Liaoning | 20210531 | Heilongjiang | 20200908 |
| S8 | Liaoning | 20210312 | Liaoning | 20210605 |
| S9 | Liaoning | 20200728 | Jilin | 20210525 |
| S10 | Jilin | 20210607 | Jilin | 20210607 |

Table. S2 Sequences of primers for RT-qPCR (rat)

| Gene | Forward primer | Reverse primer |
| --- | --- | --- |
| FXR | CCATTTACAAGCCACGGACG | CCCAGGTTGGAATAATAGGAGG |
| FOXP3 | CAGCTAAGCCTATGGCTCCTTC | GTCTGTCCTGGAGAAGTGCCTG |
| TGF-β1 | GCTGAACCAAGGAGACGGAATA | GCTGAACCAAGGAGACGGAATA |
| β-actin | GCTGAACCAAGGAGACGGAATA | GTTGGCATAGAGGTCTTTACGG |

Table. S3 Weight record for each group of rats

| Weight（kg）  Group | 1(Week) | 2(Weeks) | 3(Weeks) | 4(Weeks) |
| --- | --- | --- | --- | --- |
| Ctrl | 262.60±9.26 | 306.50±15.28 | 352.20±18.19 | 380.30±33.04 |
| Mod | 255.50±8.61 | 279.80±13.17^##^ | 302.14±18.20^##^ | 319.42±22.74^##^ |
| H-DSP | 263.00±9.36 | 294.70±24.31 | 337.56±10.25^**^ | 359.44±17.76^**^ |
| L-DSP | 268.80±14.02 | 284.50±8.96^#^ | 331.8±14.55^**^ | 351.67±31.42^*^ |
| DXM | 277.71±21.96 | 298.42±19.25 | 341.57±20.74^**^ | 365.11±27.57^**^ |

Table. S4 Linear equation and range of each bile acid standard

| Component | Linear equation | R^2^ | Linear range/ng·mL^-1^ |
| --- | --- | --- | --- |
| CA | y = 0.0027x + 0.1772 | 0.9994 | 5-5000 |
| ACA | y = 0.0019x + 0.1603 | 0.9983 | 5-5000 |
| DCA | y = 0.0003x + 0.0348 | 0.9983 | 5-5000 |
| CDCA | y = 0.0003x + 0.0471 | 0.9934 | 5-5000 |
| UDCA | y = 0.0035x + 0.3629 | 0.9972 | 5-5000 |
| LCA | y = 0.0001x + 0.0148 | 0.9946 | 5-5000 |
| TCA | y = 6×10^-5^x + 0.0029 | 0.9985 | 5-5000 |
| TDCA | y = 0.0001x + 0.008 | 0.9993 | 5-5000 |
| TCDCA | y = 0.0002x + 0.0153 | 0.9971 | 5-5000 |
| TUDCA | y = 0.0001x + 0.0069 | 0.9987 | 5-5000 |
| TLCA | y = 0.0002x + 0.0061 | 0.9990 | 5-5000 |
| GCA | y = 0.0006x + 0.0438 | 0.9992 | 5-5000 |
| GDCA | y = 0.0005x + 0.0476 | 0.9974 | 5-5000 |
| GCDCA | y = 0.0007x + 0.0439 | 0.9995 | 5-5000 |
| GUDCA | y = 0.0006x + 0.0535 | 0.9980 | 5-5000 |
| GLCA | y = 0.0007x + 0.0609 | 0.9962 | 5-5000 |

Table. S5 Precision of each bile acid in rat feces

| Component | RSD（%） | | |
| --- | --- | --- | --- |
|  | Precision (bottom) | Precision (middle) | Precision (high) |
| CA | 3.40 | 4.48 | 4.16 |
| ACA | 1.86 | 1.91 | 2.91 |
| DCA | 5.28 | 1.94 | 4.87 |
| CDCA | 11.54 | 9.38 | 2.17 |
| UDCA | 2.61 | 0.63 | 1.90 |
| LCA | 4.10 | 4.04 | 6.23 |
| TCA | 2.03 | 2.42 | 4.12 |
| TDCA | 2.86 | 2.01 | 2.16 |
| TCDCA | 2.65 | 6.44 | 1.81 |
| TUDCA | 1.83 | 2.26 | 2.38 |
| TLCA | 3.68 | 1.83 | 5.56 |
| GCA | 2.16 | 1.59 | 1.23 |
| GDCA | 3.08 | 1.58 | 2.80 |
| GCDCA | 2.95 | 1.88 | 2.94 |
| GUDCA | 4.21 | 0.83 | 3.39 |
| GLCA | 3.34 | 2.79 | 5.83 |

Table. S6 Stability of each bile acid in rat feces

| Component | Room temperature 6 h RSD（%） | | | Sampler 24 h RSD（%） | | |
| --- | --- | --- | --- | --- | --- | --- |
|  | Precision (bottom) | Precision (middle) | Precision (high) | Precision (bottom) | Precision (middle) | Precision (high) |
| CA | 1.51 | 0.79 | 1.29 | 1.44 | 2.19 | 1.95 |
| ACA | 0.98 | 0.71 | 1.57 | 3.76 | 1.29 | 0.95 |
| DCA | 7.56 | 5.36 | 2.25 | 8.80 | 2.27 | 1.31 |
| CDCA | 18.18 | 6.21 | 2.37 | 10.11 | 1.70 | 0.84 |
| UDCA | 2.67 | 1.63 | 1.90 | 1.76 | 0.94 | 4.07 |
| LCA | 11.72 | 11.36 | 5.84 | 5.22 | 6.13 | 5.76 |
| TCA | 10.95 | 2.66 | 0.06 | 5.84 | 1.88 | 2.04 |
| TDCA | 0.20 | 1.94 | 1.84 | 7.24 | 1.39 | 1.14 |
| TCDCA | 3.65 | 2.32 | 1.74 | 2.40 | 3.57 | 1.36 |
| TUDCA | 4.65 | 3.00 | 2.62 | 8.46 | 3.80 | 1.66 |
| TLCA | 3.80 | 2.16 | 0.84 | 5.30 | 2.99 | 2.46 |
| GCA | 2.51 | 0.82 | 1.42 | 5.22 | 2.12 | 1.96 |
| GDCA | 6.97 | 3.05 | 1.17 | 1.38 | 1.55 | 0.80 |
| GCDCA | 2.89 | 2.59 | 1.53 | 1.39 | 1.45 | 0.28 |
| GUDCA | 3.20 | 2.50 | 2.53 | 1.04 | 1.31 | 0.56 |
| GLCA | 2.59 | 1.06 | 1.15 | 0.94 | 1.63 | 1.42 |

Table. S7 Each bile spike recovery rate

| Component | Sample recovery (bottom) | RSD（%） | Sample recovery (middle) | RSD（%） | Sample recovery (high) | RSD（%） |
| --- | --- | --- | --- | --- | --- | --- |
| CA | 98.23 | 15.91 | 104.73 | 12.31 | 112.09 | 9.99 |
| ACA | 101.25 | 10.94 | 120.56 | 1.46 | 87.89 | 14.48 |
| DCA | 118.05 | 5.60 | 115.63 | 6.34 | 96.13 | 0.77 |
| CDCA | 97.04 | 5.41 | 106.14 | 5.93 | 96.00 | 7.01 |
| UDCA | 105.04 | 10.72 | 96.59 | 15.11 | 95.12 | 5.13 |
| LCA | 104.39 | 17.90 | 84.23 | 21.45 | 97.08 | 9.16 |
| TCA | 127.56 | 5.91 | 88.65 | 8.36 | 112.04 | 4.88 |
| TDCA | 92.91 | 10.94 | 104.58 | 7.27 | 99.54 | 15.47 |
| TCDCA | 114.78 | 13.41 | 103.56 | 5.22 | 92.15 | 18.82 |
| TUDCA | 120.69 | 2.47 | 112.10 | 3.02 | 92.34 | 3.07 |
| TLCA | 96.53 | 21.80 | 100.74 | 3.30 | 100.81 | 16.35 |
| GCA | 127.86 | 9.19 | 82.74 | 13.01 | 101.35 | 7.17 |
| GDCA | 85.35 | 7.80 | 102.64 | 11.04 | 94.46 | 5.87 |
| GCDCA | 95.65 | 11.47 | 111.84 | 8.02 | 103.93 | 8.20 |
| GUDCA | 94.42 | 17.18 | 84.28 | 5.57 | 106.04 | 18.61 |
| GLCA | 102.79 | 2.79 | 111.22 | 6.42 | 78.88 | 7.64 |
